# Volumetric Properties of Four-Stranded DNA Structures

**DOI:** 10.3390/biology10080813

**Published:** 2021-08-22

**Authors:** Tigran V. Chalikian, Robert B. Macgregor

**Affiliations:** Department of Pharmaceutical Sciences, Leslie Dan Faculty of Pharmacy, University of Toronto, 144 College Street, Toronto, ON M5S 3M2, Canada; rob.macgregor@utoronto.ca

**Keywords:** G-quadruplex, *i*-motif, volumetric properties, pressure-temperature phase diagram, thermodynamics

## Abstract

**Simple Summary:**

The volumetric properties of biomolecules define their pressure stability, while also characterizing their intrinsic and hydration properties. In this paper, we review the recent progress in volumetric investigations of G-quadruplexes and *i*-motifs, four-stranded secondary structures of DNA that have been found in the cell and implicated in regulatory genomic functions. Although the volumetric studies of G-quadruplexes and *i*-motifs are still in their nascent state, the data on volume, expansibility, and compressibility accumulated to date have begun to provide insights into the balance of forces governing the stability of these non-canonical structures. We present the available volumetric data and discuss how they can be rationalized in terms of intra-and intermolecular interactions involving G-quadruplexes and *i*-motifs including their solute-solvent interactions.

**Abstract:**

Four-stranded non-canonical DNA structures including G-quadruplexes and *i*-motifs have been found in the genome and are thought to be involved in regulation of biological function. These structures have been implicated in telomere biology, genomic instability, and regulation of transcription and translation events. To gain an understanding of the molecular determinants underlying the biological role of four-stranded DNA structures, their biophysical properties have been extensively studied. The limited libraries on volume, expansibility, and compressibility accumulated to date have begun to provide insights into the molecular origins of helix-to-coil and helix-to-helix conformational transitions involving four-stranded DNA structures. In this article, we review the recent progress in volumetric investigations of G-quadruplexes and *i*-motifs, emphasizing how such data can be used to characterize intra-and intermolecular interactions, including solvation. We describe how volumetric data can be interpreted at the molecular level to yield a better understanding of the role that solute–solvent interactions play in modulating the stability and recognition events of nucleic acids. Taken together, volumetric studies facilitate unveiling the molecular determinants of biological events involving biopolymers, including G-quadruplexes and *i*-motifs, by providing one more piece to the thermodynamic puzzle describing the energetics of cellular processes in vitro and, by extension, in vivo.

## 1. Introduction

**Figure 1:**
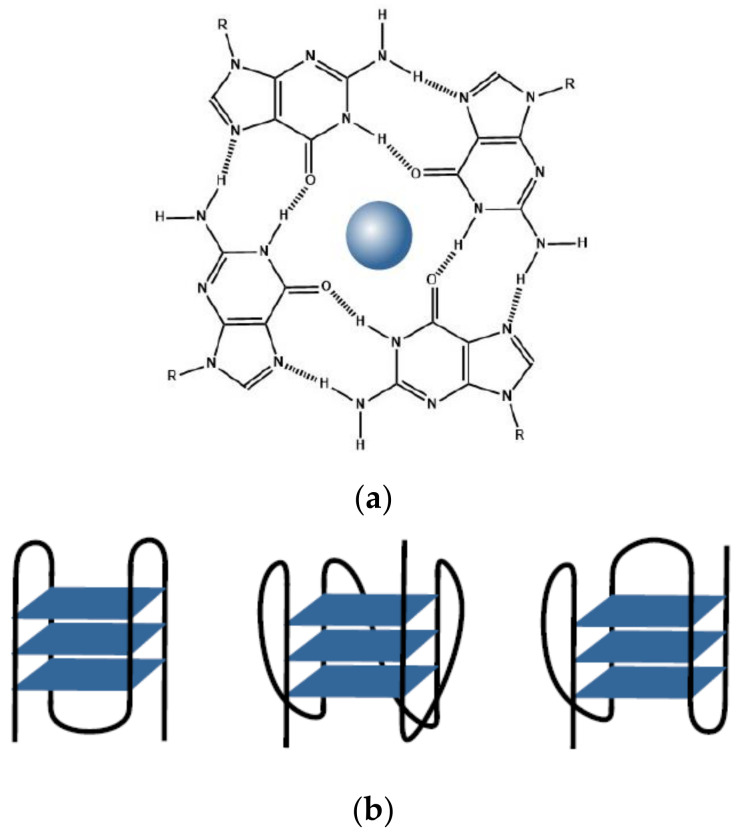
(**a**) Structure of a G-quartet with a coordinated ion; (**b**) Schematic representation of antiparallel, parallel, and hybrid intramolecular G-quadruplexes.

DNA molecules rich in guanine are prone to folding into four-stranded G-quadruplex structures, while cytosine-rich molecules tend to fold into four-stranded *i*-motif structures at slightly acidic pH [1,2,3,4,5,6,7,8,9]. G-quadruplexes are formed by stacking of two or more G-tetrads on top of each other. A G-tetrad represents a cyclic planar construct in which four guanine bases are linked together via Hoogsteen hydrogen bonds as shown in Figure 1a. The stacking results in the formation of a central cavity in which mono- or divalent cations are coordinated to the O6 atoms of guanines [1,2,3,7,10,11,12]. Sodium and potassium are the two biologically most relevant cations stabilizing G-quadruplex structures. The four consecutive G-runs involved in the formation of stacked tetrads in an intramolecular G-quadruplex are connected to each other via three single stranded linkers known as loops. The polarity of the loops defines the specific topology assumed by the G-quadruplex. G-quadruplexes can assume a parallel, antiparallel, or hybrid topologies with the molecularity ranging from mono to tetra (Figure 1b) [7,8,13].

**Figure 2:**
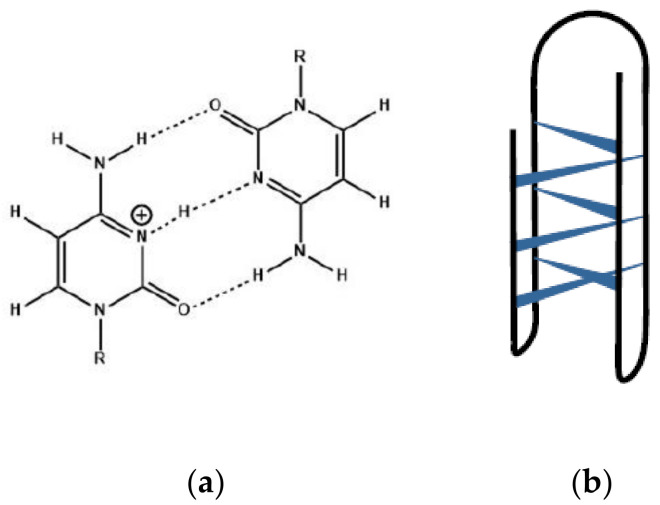
(**a**) A hemiprotonated cytosine-cytosine^+^ base pair; (**b**) Schematic representation of an *i*-motif structure.

Cytosine-rich DNA molecules can fold into the four-stranded *i*-motif conformation in which two parallel duplexes interact in an anti-parallel manner through mutual intercalation of hemiprotonated cytosine base pairs (Figure 2a,b) [4,5,6,14,15,16]. Although *i*-motif structures are favored at slightly acidic pH, they may exist at neutral pH, making the study of *i*-motifs biologically relevant [4,14,17,18,19,20]. In fact, there is increasing evidence suggesting that four-stranded nucleic acid structures, including G-quadruplex and *i*-motif structures, exist in the cell and are involved in regulation of genomic events including telomere control, gene expression, and DNA replication [10,21,22,23,24,25,26,27,28,29,30]. The existence of *i*-motifs in vivo may be related to an increase in the pK_a_ of cytosine protonation in the crowded environment of the cell [31]. In addition, given the excluded volume effect, crowders may stabilize the compact *i*-motif conformation relative the extended unfolded conformation [31].

The thermodynamic and kinetic properties of interconversions between the duplex, tetraplex, and single-stranded conformations will necessarily constrain and define the role these structures play in vivo [7,32,33]. The stability characteristics of G-quadruplex and *i*-motif structures have been extensively and systematically studied by varying parameters such as temperature, pH, salt, and the concentration of cosolvent [4,5,7,31,32,34,35,36,37,38,39,40,41,42]. These studies have provided a wealth of information about the modulation of the differential free energy of the folded and unfolded conformations of the two tetrahelical DNA structures as a function of temperature, pH, salt, and the identity and concentration of cosolvents [32]. Comparative analyses of thermodynamic data have provided valuable insights into the contributions of intra- and intermolecular interactions (e.g., counterion-DNA interactions) and individual structural features (e.g., the length and nucleotide content of loops) to the stability of G-quadruplexes and *i*-motifs [32].

The volumetric data obtained from pressure-dependent measurements complement the stability data afforded by more conventional temperature-, pH-, salt-, and cosolvent-dependent studies [43,44,45,46,47,48,49]. Analysis of the effect of hydrostatic pressure on the equilibrium between the folded and unfolded DNA structures yield molar changes in volume, expansibility, and compressibility that accompany formation of the folded structures. The volumetric properties of solutes are determined by the entire ensemble of intra- and intermolecular interactions involving the solute, including solute–solvent interactions, and volume and energy fluctuations of the solute molecule [45,46,47,50,51,52,53,54,55]. The volumetric characteristics of G-quadruplexes and *i*-motifs reflect their structural and hydration properties, which differ significantly from those of other nucleic acid secondary structures, such as the B-form DNA duplex. The four-stranded structures are globular in shape with a surface charge density lower than that of other DNA structures; in addition, the G-quadruplex exhibits a compressible and expandable internal cavity [34,35,56,57].

In this work, we describe the current state of the art and give an overview of the studies that have dealt with the volumetric characterization of G-quadruplex and *i*-motif structures. We begin by defining volumetric properties. Next, we outline the experimental methods that have been used in volumetric investigations and explain how macroscopic volumetric properties can be rationalized to gain microscopic insights. We subsequently proceed to reviewing published data on changes in volume, expansibility, and compressibility accompanying conformational transitions involving tetraplex DNA structures. Finally, we discuss the use of volumetric properties to construct the pressure–temperature diagram of G-quadruplex stability.

## 2. Definitions and Experimental Methods

### 2.1. Observables

The partial molar volume of a solute, *V°*, is the pressure slope of its chemical potential, *μ:*

(1) $$V{}^{\circ}={\left(\frac{\partial \mu }{\partial P}\right)}_{T}=\underset{C\to 0}{\lim}{\left(\frac{\partial V}{\partial N}\right)}_{T,P}$$

where *P* is the pressure; *T* is the temperature; *V* is the volume of solution; *N* is the number of moles of a solute in solution; and *C* is the concentration of a solute.

The partial molar expansibility of a solute is the temperature derivative of its partial molar volume:

(2) $$E{}^{\circ}=(\frac{\partial V{}^{\circ}}{\partial T}{)}_{P}=(\frac{\partial^{2}\mu }{\partial P\partial T})=\underset{C\to 0}{\lim}{\left(\frac{\partial \alpha V}{\partial N}\right)}_{T,P}$$

where *α* = $\frac{1}{V}{\left(\frac{\partial V}{\partial T}\right)}_{P}$ is the coefficient of thermal expansibility of solution; and *E =* *αV* is the expansibility of solution.

The partial molar isothermal compressibility of a solute is the negative pressure derivative of partial molar volume:

(3) $$K{{}^{\circ}}_{T}=(\frac{\partial V{}^{\circ}}{\partial P}{)}_{T}=-{\left(\frac{\partial^{2}\mu }{\partial P^{2}}\right)}_{T}=\underset{C\to 0}{\lim}{\left(\frac{\partial \beta_{T}V}{\partial N}\right)}_{T,P}$$

where *β_T_* = *−* $\frac{1}{V}{\left(\frac{\partial V}{\partial P}\right)}_{T}$ is the coefficient of isothermal compressibility of solution; and *K_T_ =* *β_T_V* is the isothermal compressibility of solution.

The partial molar adiabatic compressibility of a solute is given by:

(4) $$K{{}^{\circ}}_{S}=\underset{C\to 0}{\lim}{\left(\frac{\partial \beta_{S}V}{\partial N}\right)}_{T,P}$$

where *β_S_ = −*$\frac{1}{V}{\left(\frac{\partial V}{\partial P}\right)}_{S}$ is the coefficient of adiabatic compressibility of solution; *S* is the entropy; and *K_S_ =* *β_S_V* is the adiabatic compressibility of solution.

Partial molar isothermal and adiabatic compressibilities are related to each other via the relationship [58,59]:

(5) $$K{{}^{\circ}}_{T}=K{{}^{\circ}}_{S}+\frac{T\alpha_{0}^{2}}{\rho_{0}C_{P0}}\cdot \left(\frac{2E{}^{\circ}}{\alpha_{0}}-\frac{C{{}^{\circ}}_{P}}{\rho_{0}c_{P0}}\right)$$

where *α*_0_, *ρ*_0_, and *c_P_*_0_ are the coefficient of thermal expansion, density, and specific heat capacity of the neat solvent, respectively; and *C°_P_* is the partial molar heat capacity of a solute. 

According to scaled particle theory, the partial molar volume, *V°*, can be broken down into the following terms [60,61]:

*V°* = *V_C_* + *V_I_* + *β_T0_RT* (6)

where *V_C_* is the volume of the cavity comprising a solute; it is given by *V_C_ = V_M_ + V_T_; V_M_* is the molecular volume of a solute; *V_T_* is the void volume around a solute (the thermal volume); *V_I_* is the interaction volume that is a change in solvent volume under the influence of solute-solvent interactions; and *β_T_*_0_ is the coefficient of isothermal compressibility of solvent.

Interaction volume, *V_I_* is related to the properties of water of solute hydration via *V_I_ = n_h_(V°_h_ − V°*_0_*)*, where *n_h_* is the hydration number (the number of water molecules influenced by the solute), and *V°_h_* and *V°*_0_ are the partial molar volumes of water of hydration and bulk water, respectively.

The partial molar expansibility, *E°*, and adiabatic compressibility, *K°_S_*, of a solute are related to its intrinsic and hydration properties as follows:

*E° = E_M_ + n_h_(E°_h_ − E°_0_)* (7)

*K°_S_ = K_M_ + n_h_(K°_Sh_ − K°_S0_)* (8)

where *E_M_* and *K_M_* are, respectively, the intrinsic expansibility and compressibility of the solute molecule; *E°_h_* and *E°*_0_ are the partial molar expansibilities of water of hydration and bulk water, respectively; and *K°_Sh_* and *K°_S_*_0_ are the partial molar adiabatic compressibilities of water of hydration and bulk water, respectively.

The volumetric properties of a solute can be expressed more rigorously based on the concepts of statistical thermodynamics in which water of hydration is represented by a heterogeneous network of solvent molecules with varying affinities for the solute [62,63,64]. The statistical thermodynamic formalism has been extended to the analysis of the volumetric properties of solutes in binary solvents consisting of water and water-miscible cosolvents [51,65].

### 2.2. Experimental Techniques

Differential measurements of density of solution and solvent have been widely employed to measure the partial molar volume, *V°*, of solutes and changes in volume, Δ*V*, accompanying their binding events and conformational transitions [66,67,68,69,70,71,72,73,74,75,76,77]. The partial molar volume, *V°*, of a solute can be determined from density data as follows:

(9) $$V{}^{\circ}=\frac{M}{\rho_{0}}-\frac{\rho -\rho_{0}}{\rho_{0}C}=\frac{M}{\rho }-\frac{\rho -\rho_{0}}{\rho \rho_{0}m}$$

where *ρ* and *ρ*_0_ are the densities of solution and solvent, respectively; *M* is the molecular mass of a solute; and *C* and m are the molar and molal concentrations of a solute, respectively. 

If measurements of volume are carried out as a function of temperature, the resulting data can be used to determine the partial molar expansibility, *E°*, of a solute or a change in expansibility, Δ*E*, accompanying a reaction involving a solute such as a conformational transition or ligand binding [78]. Pressure-perturbation calorimetry (PPC) offers an alternative way to determine the partial molar expansibility, *E°*, of a solute as a function of temperature [79,80,81,82,83]. If a change in temperature within the experimental range causes a conformational transition of the solute, the measured *E°(T)* profile will display a characteristic peak. Given *E° = (*$\frac{\partial V{}^{\circ}}{\partial T}$*)_P_* [see Equation (2)], a change in volume, Δ*V*, accompanying the conformational transition equals the area under the peak:

(10) $$\Delta V=\int_{T_{1}}^{T_{2}}E^{o}\left(T\right)dT$$

A combination of density and sound velocity measurements can be used to determine the partial molar adiabatic compressibility, *K°_S_*, of solutes and changes in adiabatic compressibility, Δ*K_S_*, accompanying binding events and conformational transitions [84,85]. Sound velocity, *U*, in a medium is related to its density, *ρ*, and coefficient of adiabatic compressibility, *β_S_*, via the Newton-Laplace equation: *U*^2^ *=* (*ρβ_S_*)^−1^. Differentiation of this equation with respect to the concentration of a solute yields the following relationship for its partial molar adiabatic compressibility for the limit of infinite dilution [84,86,87]:

(11) $$K{{}^{\circ}}_{S}=\beta_{S0}\left(2V{}^{\circ}-2\left[U\right]-\frac{M}{\rho_{0}}\right)$$

where *β_S_*_0_ is the coefficient of adiabatic compressibility of solvent; *[U] =* $\frac{U-U_{0}}{U_{0}C}$ is the relative molar sound velocity increment of a solute; *U* and *U*_0_ are the sound velocities in solution and solvent, respectively.

Pressure-dependent measurements of conformation-sensitive spectroscopic parameters (typically, light absorption, fluorescence, NMR, and, more recently, circular dichroism) have been used to monitor pressure-induced shifts in the conformational equilibria of proteins and nucleic acids [88,89,90,91]. Provided that the solute population is restricted to two conformations (folded and unfolded), such measurements enable one to determine the equilibrium constant, *K*, as a function of pressure. In turn, the pressure dependence of *K* can be used to calculate a change in volume, Δ*V*, accompanying the conformational transition:

(12) $$\Delta V=-RT{\left(\frac{\partial lnK}{\partial P}\right)}_{T}$$

Alternatively, Δ*V* can be determined from the Clausius–Clapeyron relation:

(13) $$\frac{dT_{M}}{dP}=T_{M}\frac{\Delta V}{\Delta H}$$

where *T_M_* is the transition temperature; and Δ*H* is the transition enthalpy.

## 3. Differential Volume of Four-Stranded and Single-Stranded Conformations

### 3.1. G-quadruplexes

Table 1 presents a compilation of literature data on changes in volume accompanying unfolding transitions of G-quadruplexes differing in topology and sequence. Inspection of Table 1 reveals that G-quadruplex-to-single strand transitions are accompanied by negative changes in volume, Δ*V* [48,92,93,94,95,96,97]. To rationalize a change in volume accompanying G-quadruplex unfolding, one needs to carefully consider the entire set of molecular interactions that may contribute to the change. Equation (6) can be modified to analyze the molecular origins underlying the negative values of Δ*V* observed for G-quadruplex unfolding:

*ΔV = ΔV_M_ + ΔV_T_ + ΔV_I_ + n_M+_V_M+_* (14)

where *n_M+_* is the number of stabilizing cations released from the central cavity to the bulk; and *V_M+_* is the partial molar volume of the stabilizing cation.

The differential molecular, Δ*V_M_*, and thermal, Δ*V_T_*, volumes of the G-quadruplex and coiled states of DNA can be computed from the X-ray or NMR structure of the G-quadruplex conformation and the molecular dynamics-simulated single-stranded conformations. A change in thermal volume, Δ*V_T_*, is related to a change in solvent-accessible surface area, Δ*S_A_*, via Δ*V_T_ =* δΔ*S_A_*, where δ *=* 0.5 Å is the thickness of the thermal volume [98].

Table 2 shows the computed changes in intrinsic (molecular) volume, Δ*V_M_*, and solvent accessible surface area, Δ*S_A_*, associated with the unfolding transitions of three G-quadruplex structures, specifically, the Na^+^-stabilized antiparallel human telomeric G-quadruplex Tel22 [d(A(G_3_T_2_A)_3_G_3_)], the K^+^-stabilized hybrid human telomeric G-quadruplex Tel26 [d(A_3_(G_3_T_2_A)_3_G_3_A_2_)], and the K^+^-stabilized parallel c-MYC G-quadruplex [d(TGAG_3_TG_3_TAG_3_TG_3_T_2_)]. It is tempting to ascribe the negative change in volume to the presence of the intramolecular cavity within the G-quadruplex, which makes it distinct from other secondary structures (e.g., double- and triple-stranded DNA). However, elimination of the central cavity due to unfolding provides just one of the negative contributions to the change in volume. This source of negative volume change must be considered in concert with other contributions such as the release of counterions and changes in hydration and thermal volume.

The release of cations internally bound inside the central cavity contributes to the volumetric properties of G-quadruplex transitions. The *n_M+_V_M+_* term in Equation (14) serves to take this contribution into account. On the other hand, G-quadruplex unfolding is not accompanied by a pronounced release of externally bound (condensed) counterions [34,35,56]. In this respect, G-quadruplexes are distinct from other DNA structures, such as the double-stranded B-DNA. Depending on the specific G-quadruplex topology and loop and flanking sequences, the unfolding transition may lead to a very slight release, no release, or significant uptake of external counterions [34,35,56]. Thus, the uptake or release of these counterions should contribute only modestly to the observed changes in volume.

Inspection of data in Table 2 reveals a change in molecular volume, Δ*V_M_*, which involves the contribution due to the elimination of the central cavity, is not the main component of the experimentally measured change in volume accompanying the unfolding transitions of G-quadruplexes. The thermal, Δ*V_T_*, and interaction, Δ*V_I_*, contributions are large and play a decisive role in determining the magnitude and the sign of the overall change in volume, Δ*V*, associated with G-quadruplex unfolding.

Changes in volume, Δ*V*, accompanying G-quadruplex unfolding transitions have been combined with structural data on Δ*V_M_* and Δ*S_A_* to estimate the values of Δ*V_I_* and changes in hydration, Δ*n_h_* [92,93]. The estimates of Δ*n_h_* were 103, 432, and 170 for the Tel22, Tel26, and c-MYC G-quadruplexes, respectively [92,93]. Thus, G-quadruplex-to-coil transitions are all accompanied by an increase in hydration with a considerable uptake of water from the bulk. The estimated changes in hydration represent the differential hydration of the folded and unfolded states. A recent crystallographic study revealed elaborate, topology-dependent, organized networks of water molecules in the grooves and loop regions of G-quadruplexes [100]. It was found that the primary sphere water molecules make direct contacts with groove and loop atomic groups, thereby contributing to the stability of the specific G-quadruplex topology and loop conformations [100]. Disruption of such water networks contributes to the changes in hydration that are observed in and evaluated from the results of volumetric measurements.

### 3.2. Influence of the Bases in the Loops

The Sugimoto and Macgregor groups simultaneously published systematic studies of the extent to which the loops of G-quadruplex structures influence the effect of hydrostatic pressure on the stability of these tetrahelical structures [97,101]. The experimental space in such measurements is very large; for each simple, monomolecular oligonucleotide that can fold into a G-quadruplex, there are three different loops, each of which can have a different sequence and number of bases (or other linking moieties). In this respect, loops are highly polymorphic; and we are still a long way from an understanding of how the loops influence molar changes in volume that accompany folding of intramolecular G-quadruplexes.

Two systems have been studied; these are the thrombin binding aptamer (TBA) and the human telomeric sequence (Tel22) [97,101]. In order to attempt to isolate the factors that arise solely from the loops, the sequence of the loops has been systematically altered while preserving the topology of the folded structure [97,101]. The original and altered G-quadruplex-forming oligonucleotides that have been investigated are shown in Table 3 and Table 4. In addition to nucleic acid base substitutions, Takahashi and Sugimoto examined the behavior of TBA derivatives in which the loops were replaced by a 12-carbon methylene linker, (-CH_2_-)_12_ [101]. These substitutions remove the ability of the bases in the loops to stack with the G-tetrads in TBA.

Inspection of the data in Table 3 and Table 4 reveals that the response of the TBA- and Tel22-based systems to hydrostatic pressure is qualitatively similar. The unfolding transitions of the studied G-quadruplexes shift to lower temperatures with increasing pressure regardless of the sequence of the loops, even when a (-CH_2_-)_12_ linker substitutes the bases of the loops; changes in volume, Δ*V*, accompanying unfolding of the G-quadruplexes are all negative. However, the magnitude of Δ*V* depends on the nature of the substitution. In some cases, the substitution leads to a greater pressure sensitivity, while, in other cases, it does not result in an appreciable change in Δ*V*. Note that Takahashi and Sugimoto [101] reported their Δ*V* values at the *T_M_* of G-quadruplex unfolding, whereas the data reported by Li et al. [97] were extrapolated to a common temperature of 57 °C (near the transition temperatures). Although the *T_M_* values of the G-quadruplexes in the two studies appear to be similar, a more accurate picture of the role of the loops in the volume change of G-quadruplex unfolding would emerge if the values of Δ*V* from the two studies are extrapolated to the same temperature.

Takahashi and Sugimoto have also studied the pressure dependence of the stability of the TBA-based G-quadruplexes in aqueous solutions containing ethylene glycol, PEG200, and PEG4000 [94,95,101]. The values of Δ*V* for the TBA G-quadruplex and its derivatives in the presence of PEG200 are shown in Table 3. The purpose of including cosolvents is to study the role of solvation of the loops in the observed pressure dependences. Independent of the type of the cosolvent, the effect of pressure on the stability was significantly reduced in solutions containing cosolvents relative to the values of Δ*V* obtained in an aqueous buffer. For example, a change in volume, Δ*V*, accompanying the unfolding of TBA G-quadruplex decreases from −54.6 cm^3^ mol^−1^ in water to −12.9 cm^3^ mol^−1^ in PEG200 [94]. This finding was rationalized in terms of changes in interaction volume, Δ*V_I_*, in Equation (14); it appears that the effect of the loop sequence on the molar changes in volume arises predominantly from the differential hydration of the loops.

It is apparent that other factors such as topological differences (i.e., parallel, anti-parallel, etc.) may also contribute to the observed behavior. In addition, it seems reasonable to propose that the stacking of the G-tetrads or the stacking of the bases in the loops with the G-tetrads might also significantly contribute to the molar volume change of unfolding. The role of the stacking of the G-tetrads has not been directly assessed to date. The data originating from the -(CH_2_)_12_-substituted oligonucleotides provide insights into how the stacking of the bases in the loops with the two terminal G-tetrads influence the volume change. Currently, however, it is difficult to assign with any degree of confidence the differential volumetric properties of the oligonucleotide constructs with and without the -(CH_2_)_12_- links to any one specific molecular interaction. Additional measurements are required in order to parse the roles of stacking and folding topology in the pressure-dependent stability of G-quadruplexes.

### 3.3. i-Motifs

Table 5 lists changes in volume, Δ*V*, measured for heat-induced and pH-induced *i*-motif-to-coil transitions. In contrast to G-quadruplexes, which are characterized by large negative changes in volume upon unfolding, *i*-motif-to-single strand transitions exhibit near-zero changes in volume [57,102,103]. Because of the small value of the volume change, the stability of *i*-motifs is nearly insensitive to hydrostatic pressure. In agreement with this expectation, the transition temperatures of the heat-induced *i*-motif-to-coil transitions do not change or change very weakly with pressure [57,102,103]. When treating the volumetric properties of *i*-motif structures derived from measurements at high pressures, it is important to account for the pressure-induced changes in the pH of the solution, as the stability of an *i*-motif critically depends on pH. It is easy to confound the resulting pH-induced change in *i*-motif stability for its pressure dependence with the resulting change in volume.

In the absence of structural data on *i*-motifs, one cannot reliably estimate the magnitude or even the sign of Δ*V_M_* in Equation (14). As mentioned above, the value of Δ*V_T_* correlates with a change in solvent-accessible surface area, *S_A_*, of the DNA associated with the *i*-motif-to-coil transition. Since the *i*-motif conformation is more compact than the unfolded conformation (Δ*S_A_* is negative), the change, Δ*V_T_*, should be negative. The magnitude and the sign of Δ*V_I_* are more difficult to assess. On the one hand, polar groups of hemiprotonated cytosine residues that are hydrogen-bonded with water in the coil state become buried within the interior of the *i*-motif, thereby diminishing the extent of solute−solvent interactions. On the other hand, the proximity of negatively charged phosphate groups within *i*-motif conformation should increase the charge density and enhance the volume-reducing effect of solute−solvent interactions, Δ*V_I_*. The experimentally measured value of Δ*V* ≈ 0 suggests a near perfect compensation between the Δ*V_M_*, Δ*V_T_*, and Δ*V_I_* terms in Equation (14).

## 4. Differential Expansibility

Table 6 presents changes in expansibility, Δ*E*, associated with G-quadruplex-to-coil transitions of the Tel22 and Tel26 telomeric sequences. The partial molar expansibilities, *E°*, of the two G-quadruplexes increase upon unfolding. The relationship for Δ*E* can be obtained by modifying Equation (7):

*ΔE = ΔE_M_ + Δn_h_(E°_h_ − E°_0_) + n_M+_E_M+_ + ΔE_rel_* (15)

where *E_M+_* is the is the partial molar expansibility of the stabilizing cation; Δ*E_rel_* is the change in the relaxation contribution, *E_rel_ = (<*Δ*H*Δ*V> − <*Δ*H><*Δ*V>)/RT^2^*; <Δ*H*> and <Δ*V*> are, respectively, the ensemble average changes in enthalpy and volume relative to a “ground state” conformation.

The intrinsic term in Equation (15) is given by Δ*E_M_ = α_MU_V_MU_ − α_MF_V_MF_*, where *α_MU_* and *α_MF_* are the intrinsic coefficients of thermal expansibility of the unfolded (coil) and folded (G-quadruplex) states, respectively; and *V_MU_* and *V_MF_* are the intrinsic volumes of the unfolded (coil) and folded (G-quadruplex) states, respectively. The intrinsic coefficient of thermal expansibility of the unfolded state, *α_MU_*, is close to zero. There are currently no data on the value of *α_MF_*; however, one would expect the intrinsic coefficient of thermal expansibility of the folded state, *α_MF_*, to be a sizeable quantity owing to the expandable central cavity. Therefore, Δ*E_M_* should be negative for G-quadruplex unfolding. In the absence of data, it seems reasonable to propose that the value of *α_MF_* is on the order of the intrinsic coefficient of thermal expansibility of globular proteins. In common with G-quadruplexes, globular proteins are characterized by potentially expandable internal voids [104,105]. The average intrinsic coefficient of thermal expansibility of globular proteins is ~1 × 10^−4^ K^−1^ [50].

The relaxation component, Δ*E_rel_*, originates from the existence of a broadly distributed isoenergetic population of unfolded conformations differing in enthalpy and volume. An increase in temperature shifts the population of coil-like conformations towards the species with a greater enthalpy, which would result in a positive or negative value of Δ*E_rel_* depending on the sign of the volume difference between the high and low enthalpy subpopulations [93].

At room temperature, all low molecular weight model compounds studied to date exhibit positive partial molar expansibilities, *E°* [106,107,108,109,110,111,112,113]. The intrinsic expansibility, *E_M_*, of low molecular weight compounds is close to zero. Hence, according to Equation (7), the positive values of *E°* of small molecules are suggestive of the positive values of the differential expansibility of water of hydration and bulk water, (*E°_h_ − E°*_0_), for all functional groups independent of their chemical nature. The hydration term in Equation (15), Δ*n_h_(E°_h_ − E°*_0_*)*, is positive given the positive values of Δ*n_h_* (water is taken up by the hydration shell of the DNA upon its G-quadruplex-to-coil transition) and (*E°_h_ − E°*_0_).

## 5. Differential Compressibility

### 5.1. G-quadruplexes

Table 6 presents changes in adiabatic compressibility, Δ*K_S_*, accompanying unfolding transitions of the Tel22, Tel26, and c-MYC G-quadruplexes. The partial molar adiabatic compressibilities, *K°_S_*, of the three G-quadruplexes all decrease upon their unfolding. The relationship for Δ*K_S_* can be obtained by modifying Equation (8):

*ΔK_S_ = ΔK_M_ + Δn_h_(K°_Sh_ − K°_S0_) + n_M+_K_SM+_ + ΔK_Srel_* (16)

where *K_SM+_* is the partial molar adiabatic compressibility of the stabilizing ion. The relaxation contribution in Equation (16) is given by *K_Srel_ = (<*Δ*V^2^> − <*Δ*V>^2^) / RT*, where <Δ*V*> is the ensemble average changes in volume relative to a “ground state” conformation. The intrinsic compressibility, *K_M_*, of a solute is given by *K_M_ = β_M_V_M_*, where *β_M_* is the intrinsic coefficient of adiabatic compressibility.

Owing to the presence of the central cavity, a G-quadruplex possesses a compressible interior that is absent in its unfolded state. In other words, the value of *β_M_* of the native G-quadruplex is sizeable, but reduces to near zero in the unfolded state, which is devoid of a compressible interior. There are no estimates of the value of *β_M_* for G-quadruplexes. However, given the structural similarity of G-quadruplexes and globular proteins, the *β_M_* of a G-quadruplex can be expected to be on the order of 25 × 10^−6^ bar^−1^, the average coefficient of adiabatic compressibility of a globular protein [46,50,114,115,116]. Further studies, particularly, the pressure-dependent NMR characterization of native G-quadruplexes, may help evaluate the value of $\beta_{M}=-\frac{1}{V_{M}}{\left(\frac{\partial V_{M}}{\partial P}\right)}_{T}$, which, in turn, would assist in a more reliable estimate of the molecular determinants of changes in compressibility accompanying conformational transitions involving G-quadruplexes.

For nucleic acids, the hydration term, Δ*n_h_(K°_Sh_ − K°_S_*_0_*)*, in Eq.(16) is negative given the positive sign of Δ*n_h_* and the negative sign of (*K°_Sh_ − K°_S_*_0_) [44,47,117,118,119]. The volumetric properties of water of hydration of nucleic acids are dominated by the hydration of charged and clustered polar groups, which exhibit partial molar adiabatic compressibilities, *K°_Sh_*, of water of hydration lower than that of bulk water, *K°_S_*_0_ [45,120]. In contrast, isolated polar groups and nonpolar groups may exhibit partial molar compressibilities that are greater than that of bulk water [45,120]. Currently, it is difficult to come up with a reliable estimate of the value of (*K°_Sh_ − K°_S_*_0_) for parsing the measured changes in compressibility, Δ*K_S_*, in terms of specific components using Equation (16). For charged solutes, such as DNA, the partial molar adiabatic compressibility, *K°_Sh_*, of water of hydration is ~25% smaller than that of bulk water [64]. However, the functional groups that become solvent exposed and subsequently solvated when a G-quadruplex unfolds are not all charged. In contrast, many of them are polar uncharged or even nonpolar; for example, the functional groups of nucleic acid bases that, due to their participation in base pairing and stacking interactions in the folded state, are shielded from the solvent, but become solvent-exposed and hydrated when the structure unfolds. Consequently, there is no clarity about the specific value of (*K°_Sh_ − K°_S_*_0_) that should be used in the analysis.

The relaxation component, Δ*K_rel_*, originates from the broadly distributed ensemble of nearly isoenergetic unfolded single-stranded conformations that vary in volume. An increase in pressure shifts the ensemble of unfolded conformations towards the species with smaller volumes that gives rise to an additional positive contribution to the observed change in compressibility associated with G-quadruplex unfolding [93].

### 5.2. i-Motifs

There is only a single work that reports a change in adiabatic compressibility, Δ*K_S_*, accompanying the helix-to-coil transition of an *i*-motif [57]. For the c-MYC *i*-motif, the value of Δ*K_S_* for the pH-induced helix-to-coil transition at 25 °C is nearly zero (see Table 5) [57].

As mentioned above, the intrinsic compressibility term, *K_M_*, in Equation (16) represents the compressibility of the water-inaccessible interior core of the solute. While the intrinsic compressibility, *K_M_*, of the ensemble of unfolded conformations is close to zero, that of the folded *i*-motif conformation is less certain and, in principle, may be significant. The sign and the magnitude of hydration contribution to compressibility in Equation (16), Δ*n_h_(K_h_ − K*_0_*)*, is also unclear. The differential partial molar compressibility of hydration and bulk water, (*K_h_ − K*_0_), is negative for the unfolded, coil-like state. On the other hand, in the folded *i*-motif conformation, the value of (*K_h_ − K*_0_) may be more negative relative to the coil state (because the negatively charged phosphates are brought closer together), it may be the same, or it may be less negative (e.g., due to a more effective mutual neutralization of the positively charged cytosines and negatively charged phosphates). Should the change in intrinsic compressibility, Δ*K_M_*, in Equation (16) be near zero, that is the interior of the *i*-motif conformation is rigid, the observation that Δ*K_S_* ≈ 0 suggests the similarity of the hydration of the *i*-motif and coil states with ΔΔ*K_h_* ≈ 0. Although the current data do not enable us to discriminate between the various scenarios, the observed Δ*K_S_* ≈ 0 is an indication of a near-perfect offsetting of the Δ*K_M_* and ΔΔ*K_h_* terms in Equation (16). In addition, it should be noted that there may be a relaxation component in analogy with G-quadruplexes.

In the aggregate, the current volumetric data on *i*-motifs collected to date suggest that fortuitous compensations between the intrinsic and hydration contributions to volume and compressibility result in Δ*V* and Δ*K_S_* of zero. A compressibility change of zero implies that the compensations leading to Δ*V* being zero are not restricted to ambient pressure, but also act at elevated pressures.

## 6. Pressure-Temperature Phase Diagram

The stability of a G-quadruplex (or any other biopolymer) as a function of temperature and pressure can be presented analytically as follows [121,122,123,124,125,126]:

(17) $$\Delta G\left(P,T\right)=\Delta H_{M}\left(1-\frac{T}{T_{M}}\right)+\Delta C_{P}\left(T-T_{M}-Tln\frac{T}{T_{M}}\right)+\left[\Delta V\left(T_{R}\right)+\Delta E\left(T-T_{R}\right)\right]\left(P-P_{R}\right)-0.5\Delta K_{T}\left(P^{2}-P_{R}{}^{2}\right)$$

where Δ*H_M_* is the differential enthalpy of the folded and unfolded states at the transition temperature, T_M_, and reference pressure, *P_R_*; Δ*C_P_* is the differential heat capacity of the folded and unfolded states; Δ*V(T_R_)* is the differential volume of the folded and unfolded states at the reference temperature, *T_R_*, and pressure, *P_R_*; and Δ*E* and Δ*K_T_* are, respectively, the differential expansibility and isothermal compressibility of the folded and unfolded states.

The relationship for pressure-temperature phase diagram can be derived by equating Equation (17) to zero and solving it with respect to the denaturation pressure, *P_M_*:

(18) $$\Delta H_{M}\left(1-\frac{T}{T_{M}}\right)+\Delta C_{P}\left(T-T_{M}-Tln\frac{T}{T_{M}}\right)+\left[\Delta V\left(T_{R}\right)+\Delta E\left(T-T_{R}\right)\right]\left(P-P_{R}\right)-0.5\Delta K_{T}\left(P^{2}-P_{R}{}^{2}\right)=0$$

The reference pressure, *P_R_*, is generally set equal to ambient pressure (1 bar). It can be ignored relative to pressures, *P_M_*, at which proteins and nucleic acids denature. The latter are, typically, on the order of ~1 kbar and higher:

(19) $$0.5\Delta K_{T}P_{M}{}^{2}-\left[\Delta V\left(T_{R}\right)+\Delta E\left(T-T_{R}\right)\right]P_{M}-\Delta H_{M}\left(1-\frac{T}{T_{M}}\right)-\Delta C_{P}\left(T-T_{M}-Tln\frac{T}{T_{M}}\right)=0$$

Solving Equation (19) with respect to *P_M_*, one obtains the following:

(20) $$P_{M}=\frac{\Delta V\left(T_{R}\right)+\Delta E\left(T-T_{R}\right)\pm \sqrt{D}}{\Delta K_{T}}$$

where $D={\left[\Delta V\left(T_{R}\right)+\Delta E\left(T-T_{R}\right)\right]}^{2}+2\Delta K_{T}\left[\Delta H_{M}\left(1-\frac{T}{T_{M}}\right)-\Delta C_{P}\left(T-T_{M}-Tln\frac{T}{T_{M}}\right)\right]$.

Equation (20) has been used to compute the pressure-temperature phase diagram of the c-MYC G-quadruplex [99]. In the computation, the change in heat capacity, Δ*C_P_*, has been evaluated based on the results reported by Majhi et al. [127].

**Figure 3:**
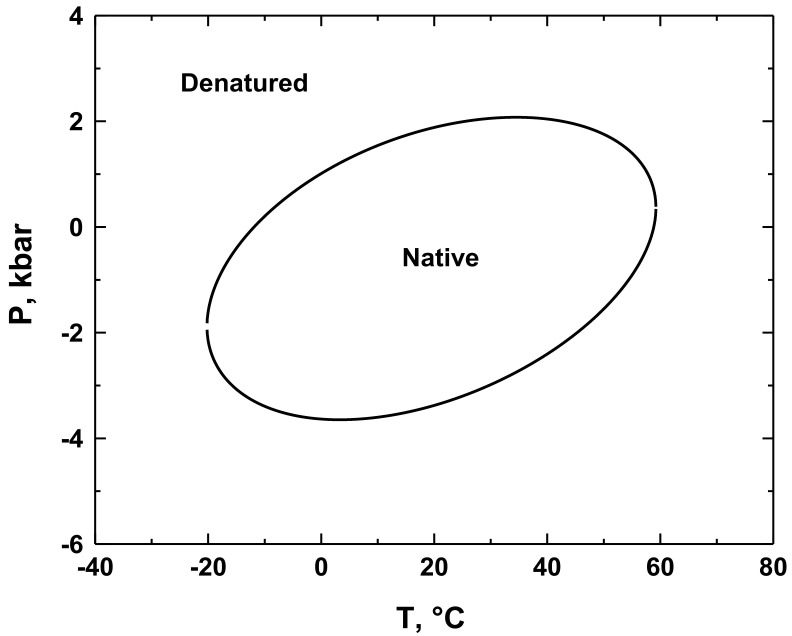
The pressure-temperature phase diagram for the stability c-MYC G-quadruplex at 50 mM CsCl and 0.1 mM KCl computed with Equation (20) from ref. [99].

Figure 3 presents the pressure–temperature stability phase diagram for the c-MYC G-quadruplex [99]. As is seen from Figure 3, the diagram is elliptic. It resembles that of a globular protein [121,122,124,128], while being distinct from that of duplex DNA [129]. We propose that the similarity of the pressure–temperature stability phase diagram of a G-quadruplex and a globular protein reflects their shared structural and volumetric features. In particular, both G-quadruplexes and globular proteins are compact and characterized by compressible intramolecular voids. The order-disorder transitions of both structures are accompanied by an increase in expansibility, Δ*E*, and reductions in volume, Δ*V*, and compressibility, Δ*K_T_*.

Pressure-induced structural changes in nucleic acid structures and the resulting inhibition of genomic processes may contribute to pressure-induced cell death and injury in microorganisms. We have previously suggested that the fine-tuned, differential pressure sensitivity of the duplex, G-quadruplex, and *i*-motif conformations adopted by specific genomic loci may be involved in regulation of genomic processes in barophilic organisms thereby governing their survival at high pressures [57]. The differential stability phase diagram of G-quadruplex and duplex DNA [129] may provide an additional platform for developing this hypothesis.

## 7. Conclusions

There is only a decade of volumetric studies of G-quadruplex and *i*-motif structures. In some ways, our understanding of the volumetric characteristics of these four-stranded structures is reminiscent of the situation with the volumetric studies of proteins in the 1980s. Despite their scarcity, the volumetric data accumulated so far have established several regularities that are common for each of these four-stranded structures. In particular, all G-quadruplex-to-coil transitions studied to date are accompanied by negative changes in volume, Δ*V*, and compressibility, Δ*K_S_*, and positive changes in expansibility, Δ*E*. The few *i*-motif-to-coil transitions studied are accompanied by near zero changes in volume, Δ*V*, and compressibility, Δ*K_S_*. While it is still difficult to reliably rationalize volumetric observations in terms of intrinsic and hydration contributions, they establish an experimental framework for deriving the pressure–temperature stability diagrams of tetraplex DNA structures. Further studies involving a wide range of G-quadruplex and *i*-motif structures are needed to understand the generality and molecular origins of these results.

## Figures and Tables

**Table 1:** Changes in volume, Δ*V*, accompanying the unfolding transitions of G-quadruplexes differing in sequence, topology, and stabilizing cation determined at temperature, *T*.

Sequence (DNA)	Topology	Cation	*T,* °C	Δ*V*, cm^3^ mol^−1^
d(G_2_T_2_G_2_TGT-G_2_T_2_G_2_) (TBA) ^a^	Antiparallel	K^+^	58.1 ± 1.4	−54.6 ± 4.2
d[A(G_3_T_2_A)_3_G_3_](Tel22) ^b^	Antiparallel	Na^+^	54.6 ± 0.9	−38.4 ± 10.1
d[A(G_3_T_2_A)_3_G_3_](Tel22) ^c^	Antiparallel	Na^+^	40.0 ± 0.6	−66 ± 3
d[A(G_3_T_2_A)_3_G_3_](Tel22) ^b^	Hybrid	K^+^	64.6 ± 2.2	−42.7 ± 6.7
d[TGA(G_3_TG_3_TA)_2_A](c-MYC) ^d^	Parallel	K^+^	83.4 ± 1.1	−16.9 ± 1.8
d(AG_3_AG_3_CGCTG_3_-AG_2_AG_3_) (KIT) ^d^	Parallel	K^+^	58.5 ± 0.4	−6.2 ± 0.9
d(T_2_G_4_CG_3_C_2_G_5_C-G_4_T_2_) (VEGF) ^d^	Parallel	K^+^	78.8 ± 1.1	−18.1 ± 4.6
d[A_3_(G_3_T_2_A)_3_G_3_A_2_](Tel26) ^e^	Hybrid	K^+^	25.0	−69 ± 7
d[TGA(G_3_TG_3_TA)_2_A](c-MYC) ^f^	Parallel	K^+^	25.0	−34 ± 15

^a^ from ref. [94]; ^b^ from ref. [97]; ^c^ from ref. [92]; ^d^ from ref. [96]; ^e^ from ref. [93]; ^f^ from ref. [99].

**Table 2:** Changes in volume, Δ*V* (cm^3^ mol^−1^), determined at 25 °C, intrinsic volume, Δ*V_M_* (cm^3^ mol^−1^), solvent accessible surface area, Δ*S_A_ (*Å^2^*)*, thermal volume, Δ*V_T_* (cm^3^ mol^−1^), and interaction volume, Δ*V_I_*, accompanying G-quadruplex unfolding transitions.

G-Quadruplex	Δ*V*	Δ*V_M_*	Δ*S_A_*	Δ*V_T_ =* δΔ*S_A_*	Δ*V_I_^a^*
Tel22	−67	−233 ^b^	1230 ^b^	370	−186
Tel26	−69	−4 ^c^	2348 ^c^	707	−779
c-MYC	−34	2 ^d^	879 ^d^	265	−306

^a^ Δ*V_I_ =* Δ*V − (*Δ*V_M_ +* Δ*V_T_ + n_M+_V_M+_)*; ^b^ computed based on PDB entry 143D [92]; ^c^ computed based on PDB entry 2HY9 [93]; ^d^ computed based on PDB entry 1XAV [34].

**Table 3:** Molar chanes in volume (Δ*V*) accompanying the unfolding transitions of thrombin binding aptamer (TBA) and thrombin binding aptamers with altered loops determined from the pressure dependences of the unfolding temperature, *T_M_*. The values were calculated from analysis of the effect of hydrostatic pressure on the thermal unfolding of the G-quadruplex using the Clapeyron equation. For TBA 1LC12 and TBA 2LC12, a 12-carbon methylene chain replaces the nucleobases in the respective loops. The changes in the original TBA sequence are underlined.

DNA	Sequence	*T_M_*, °C ^e^	Δ*V*, cm^3^ mol^−1^
TBA ^a,c^	d(G_2_T_2_G_2_TGTG_2_T_2_G_2_)	52.6 ± 3.4	−54.6 ± 4.2
TBA T3A ^b,c^	d(G_2_ATG_2_TGTG_2_T_2_G_2_)	45.1 ± 0.1	−75.5 ± 2.2
TBA G8T ^b,c^	d(G_2_T_2_G_2_TTTG_2_T_2_G_2_)	47.1 ± 6.6	−41.1 ± 2.4
TBA 1LC12 ^b,c^	d[G_2_-(CH_2_)_12_-G_2_TGTG_2_T_2_G_2_]	49.0 ± 3.9	−57.8 ± 8.4
TBA 2LC12 ^b,c^	d[G_2_T_2_G_2_-(CH_2_)_12_-G_2_T_2_G_2_]	36.5 ± 0.6	−103.4 ± 8.0
TBA ^a,d^	d(G_2_T_2_G_2_TGTG_2_T_2_G_2_)	59.3 ± 2.3	−12.9 ± 0.9
TBA T3A ^b,d^	d(G_2_ATG_2_TGTG_2_T_2_G_2_)	56.7 ± 2.2	−14.7 ± 4.9
TBA G8T ^b,d^	d(G_2_T_2_G_2_TTTG_2_T_2_G_2_)	54.5 ± 0.7	−13.2 ± 2.1
TBA 1LC12 ^b,d^	d[G_2_-(CH_2_)_12_-G_2_TGTG_2_T_2_G_2_]	56.6 ± 6.0	−9.7 ± 5.2
TBA 2LC12 ^b,d^	d[G_2_T_2_G_2_-(CH_2_)_12_-G_2_T_2_G_2_]	62.4 ± 0.3	−5.6 ± 1.7

^a^ from ref. [94]; ^b^ from ref. [101]; ^c^ the solutions contained 30 mM Tris-HCl at pH 7.0 and 100 mM KCl; ^d^ the solutions contained 30 mM Tris-HCl at pH 7.0, 100 mM KCl, and 40 wt% PEG200; ^e^ the unfolding transition at atmospheric pressure.

**Table 4:** Volume changes associated with the unfolding of the original and modified human telomeric G-quadruplexes in the presence of Na^+^ or K^+^ at 57 °C, Δ*V*_57_ (cm^3^ mol^−1^), presented by Li et al. [97]. The changes to the original Tel22 sequence are underlined. Values were obtained by analyzing the effect of hydrostatic pressure on the equilibrium between the folded and unfolded states of the respective G-quadruplexes assuming a two-state monomolecular reaction. The values were extrapolated to a common temperature of 57 °C which corresponds to the average *T_M_* of the G-quadruplexes studied. All samples contained 10 mM Tris at pH 7.4, 0.1 mM EDTA, and either 100 mM NaCl or 100 mM KCl.

DNA	Sequence	(Δ*V*, cm^3^ mol^−1^)	
		Na^+^	K^+^
Tel22	d(AG_3_T_2_AG_3_T_2_AG_3_T_2_AG_3_)	−38.4 ± 10.1	−42.7 ± 6.7
L1AAT	d(AG_3_AATG_3_T_2_AG_3_T_2_AG_3_)	−29.4 ± 5.6	−38.0 ± 7.3
L2AAT	d(AG_3_T_2_AG_3_AATG_2_T_2_AG_3_)	−29.8 ± 10.1	−35.6 ± 8.0
L3AAT	d(AG_3_T_2_AG_3_T_2_AG_3_AATG_3_)	−34.5 ± 1.1	−27.2 ± 6.4
L1TTT	d(AG_3_TTTG_3_T_2_AG_3_T_2_AG_3_)	−35.2 ± 3.0	−35.2 ± 3.9
L2TTT	d(AG_3_T_2_AG_3_TTTG_3_T_2_AG_3_)	−26.2 ± 8.0	−30.5 ± 11.1
L3TTT	d(AG_3_T_2_AG_2_T_2_AG_3_TTTG_3_)	−38.6 ± 4.0	−21.9 ± 7.9
L1AAA	d(AG_3_AAAG_3_T_2_AG_3_T_2_AG_3_)	−37.7 ± 9.0	−37.7 ± 4.8
L2AAA	d(AG_3_T_2_AG_3_AAAG_3_T_2_AG_3_)	−30.2 ± 13.1	−37.8 ± 1.0
L3AAA	d(AG_3_T_2_AG_3_T_2_AG_3_AAAG_3_)	−41.4 ± 6.8	−31.7 ± 1.0

**Table 5:** Changes in volume, Δ*V*, and adiabatic compressibility, Δ*K_S_*, accompanying the unfolding transitions of *i*-motif structures determined at temperature, T.

Sequence (DNA)	pH	*T*, °C	Δ*V*, cm^3^ mol^−1^	Δ*K_S_*, 10^−4^ cm^3^ mol^−1^bar^−1^
d(C_3_TA_2_)_3_C_3_ (Tel22-iM) ^a^	4.6	36	~0	
d(C_3_TA_2_)_3_C_3_ (Tel22-iM) ^b^	5.15	45.5	−11 ± 2	
d(T_2_AC_3_AC_3_TAC_3_A-C_3_TCA) (c-MYC-iM) ^c^	5.0	25.0	~0	~0

^a^ from ref. [102]; ^b^ from ref. [103]; ^c^ from ref. [57].

**Table 6:** Changes in adiabatic compressibility, Δ*K_S_,* and expansibility, Δ*E*, accompanying the unfolding transitions of G-quadruplexes varying in sequence, topology, and stabilizing cation at 25 °C.

Sequence (DNA)	Topology	Cation	Δ*K_S_*, 10^−4^ cm^3^ mol^−1^bar^−1^	Δ*E*, cm^3^ mol^−1^K^−1^
d[A(G_3_T_2_A)_3_G_3_](Tel22) ^a^	Antiparallel	Na^+^	−236 ± 20	0.87 ± 0.16
d[A_3_(G_3_T_2_A)_3_G_3_A_2_](Tel26) ^b^	Hybrid	K^+^	−332 ± 18	0.92 ± 0.07
d[TGA(G_3_TG_3_TA)_2_A](c-MYC) ^c^	Parallel	K^+^	−304 ± 26	

^a^ from ref. [92]; ^b^ from ref. [93]; ^c^ from ref. [99].

## Data Availability

Not applicable.
